# Five New Phenolic Compounds with Antioxidant Activities from the Medicinal Insect *Blaps rynchopetera*

**DOI:** 10.3390/molecules22081301

**Published:** 2017-08-04

**Authors:** Huai Xiao, Tian-Peng Yin, Jian-Wei Dong, Xiu-Mei Wu, Qing Luo, Jian-Rong Luo, Le Cai, Zhong-Tao Ding

**Affiliations:** 1 Functional Molecules Analysis and Biotransformation Key Laboratory of Universities in Yunnan Province, School of Chemical Science and Technology, Yunnan University, Kunming 650091, China; Xiaohuai10@263.net (H.X.); tpyin89@163.com (T.-P.Y.); jwdongyn@outlook.com (J.-W.D.); caile@ynu.edu.cn (L.C.); 2 Yunnan Provincial Key Laboratory of Entomological Biopharmaceutical R&D, Dali University, Dali 671000, China; wxm6865@163.com (X.-M.W.); lq690275304@163.com (Q.L.); ljrrong@vip.sina.com (J.-R.L.); 3 Zhuhai Key Laboratory of Fundamental and Applied Research in Traditional Chinese Medicine, Zunyi Medical University Zhuhai Campus, Zhuhai 519041, China

**Keywords:** *Blaps rynchopetera*, chemical constituents, phenolic, rynchopeterines A–E, antioxidant, anti-tumor

## Abstract

Five new phenolic compounds rynchopeterines A–E (**1**–**5**), in addition to thirteen known phenolics, were isolated from *Blaps rynchopetera* Fairmaire, a kind of medicinal insect utilized by the Yi Nationality in Yunnan Province of China. Their structures were established on the basis of extensive spectroscopic analyses (1D and 2D NMR, HR-MS, IR) along with calculated electronic circular dichroism method. Rynchopeterines A–E (**1**–**4**) exhibited significant antioxidant activities with IC_50_ values of 7.67–12.3 μg/mL measured by the 1,1-diphenyl-2-picrylhydrazyl (DPPH) assay. Besides, rynchopeterines B (**2**) and C (**3**) showed mild cytotoxicity against tumor cell Caco-2 and A549.

## 1. Introduction

Insects have long been utilized as medicines in the world, especially in China. Insects make up the largest and most diverse group of organisms on earth, and could serve as a vast potential medicinal resource. However, in contrast to efforts exploring the peptide and protein components present in insects, much less attention has been given to the insect derived small natural products and their biological properties. The potential roles that insect derived substances might play as lead compounds in drug discovery efforts has been largely ignored [1,2]. Fortunately, certain medicinal insects have drawn the scientists’ attention because of their beneficial health effects on humans. It was reported that a huge variety of chemical substances with potent biological activities which might have use as lead compounds in drug discovery have been isolated from insects in recent years [3].

**Figure 1:**
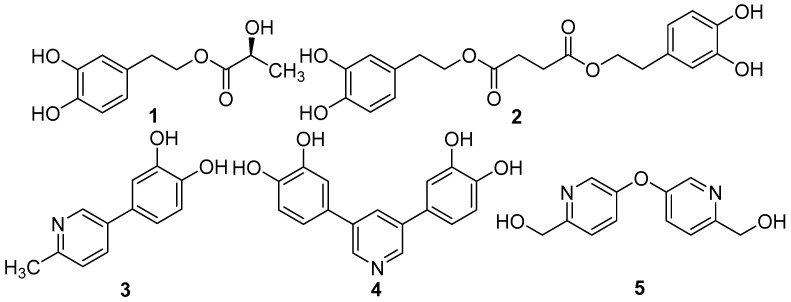
Structures of compounds **1**–**5**.

*Blaps rynchopetera* Fairmaire belongs to the family Tenebrionidae (Coleoptera), and is a kind of insect that has long been utilized as a folk medicine by the Yi Nationality in Yunnan Province of China. This insect is effective in the treatment of cough, gastritis, and various cancers [4]. Our previous studies have revealed that dipeptides and phenolics—which exhibited various pharmacological activities—are the main active ingredients of this drug [5]. As part of our continuous work on the systematic study of this insect, the chemical constituents of *B. rynchopetera* was investigated to afford five new phenolic compounds rynchopeterines A–E (**1**–**5**) (Figure 1), along with thirteen known phenolics, procatechuic acid (**6**) [6], 3,4-dihydroxyphenylacetic acid (**7**) [7], 3,4-dihydroxybenzaldehyde (**8**) [8], 3,4-dihydroxyphenylacetaldehyde (**9**) [9], methyl 3,4-dihydroxybenzoate (**10**) [10], 3,4-dihydroxyphenylacetic acid methyl ester (**11**) [11], 3,4-dihydroxyphenylacetic acid ethyl ester (**12**) [7], 2-(3,4-dihydroxyphenyl)ethanol (**13**) [12], 3,4-dihydroxyphenethylacetate (**14**) [13], blapsins A (**15**) and B (**16**) [14], 1-(3,4-dimethoxyphenyl)-4-hydroxypentan-1-one (**17**) [15], and 3,3′,4,4′-tetrahydroxybiphenyl (**18**) [16] (see Supplementary). Herein, the isolation, structural elucidation, and bioactivity of the new compounds are described.

## 2. Results

### 2.1. Structural Elucidation of Compounds ***1**–**5***

**Figure 2:**
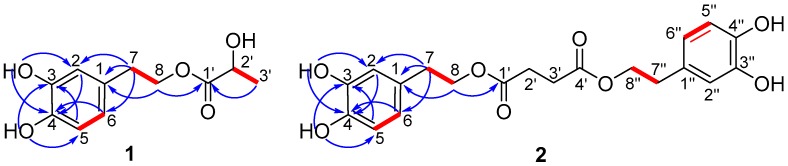
Key ^1^H-^1^H COSY (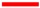) and HMBC (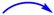) correlations of compounds **1** and **2**.

Compound **1** was isolated as a light yellow liquid, and its molecular formula was deduced to be C_11_H_14_O_5_ with an unsaturation degree of five by HR-ESIMS at *m*/*z*: 249.0738 [M + Na]^+^. The NMR spectra of **1** showed the presence of a 1,3,4-trisubstituted aromatic ring [δ_H_ 6.76 d (2.0), 6.76 d (8.0), 6.59 dd (8.0, 2.0); δ_C_ 129.5 s, 116.0 d, 145.0 s, 143.7 s, 115.2 d, 120.2 d], two phenolic hydroxyl groups (δ_H_ 7.77 brs, 7.80 brs), and two methines [δ_H_ 2.80 t (7.0), 4.21, 4.30 dt (7.0, 11.6); δ_C_ 34.2 t, 65.3 t] (Table 1), which were assigned to be a 3-hydroxytyrosol group [17]. The substituent position of the aromatic ring was further confirmed by HMBC correlations from OH-3 to C-3, OH-4 to C-4, and H-8, H-7 to C-1 (Figure 2). Besides, a lactic acid group [δ_H_ 4.23 q (7.0), 1.31 d (7.0); δ_C_ 19.9 q, 66.6 d, 174.7 s) was identified in its NMR spectra [18], which was connected to C-8 in 3-hydroxytyrosol group according to HMBC correlation from H-8 to C-1′. The absolute configuration of C-2′ was assigned to be *S* by the comparison of the theoretical electronic circular dichroism (ECD) data with the experimental spectrum (Figure 2). Two geometries were previously optimized by density functional theory (DFT) method at the B3LYP/6-31G(d) level [19]. Excitation energies and rotational strengths were calculated using time-dependent density functional theory (TDDFT) at the B3LYP/6-31G (d,p) level in acetonitrile with PCM model [20]. The ECD spectrum was simulated from electronic excitation energies and velocity rotational strengths. The results showed that the theoretical ECD data for 2′*S*-isomer was in good agreement with the experimental spectrum. Therefore, the structure of compound **1** was determined as rynchopeterine A, with its assigned NMR data listed in Table 1.

Compound **2** was isolated as light brown liquid, whose molecular formula was deduced to be C_20_H_22_O_8_ with an unsaturation degree of ten by HR-ESIMS at *m*/*z*: 413.1192 [M + Na]^+^. In combination with the MS data, two 3-hydroxytyrosol groups and an amber acid group (δ_H_ 2.59, s, 4H; δ_C_ 28.5 t, 171.8 s) were identified in its NMR spectra (Table 1) [21]. The connection of those three fragments was accomplished by the HMBC correlations from H-8 to C-1′, and from H-8″ to C-5′ (Figure 1). Hence, the structure of compound **2** was determined, and its assigned NMR data are listed in Table 1.

**Figure 3:**
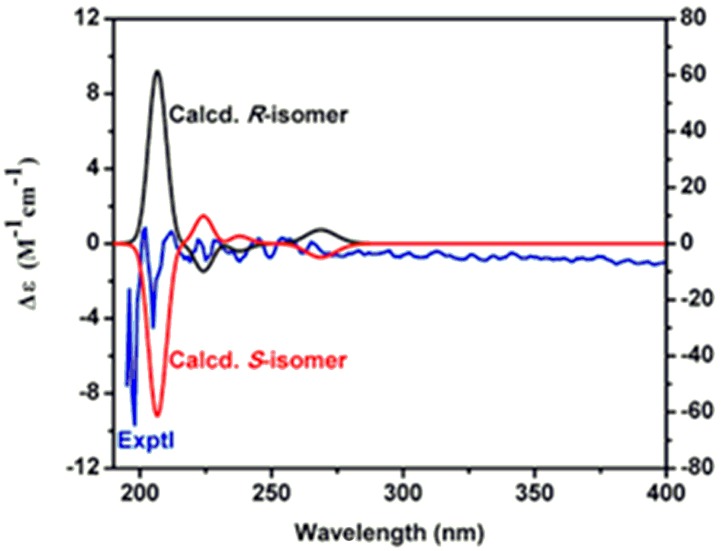
Calculated and experimental electronic circular dichroism (ECD) spectra of compound **1**.

Compound **3** was isolated as a white powder, whose molecular formula was deduced to be C_12_H_11_NO_2_ with an unsaturation degree of six by HR-ESIMS at *m*/*z*: 202.0859 [M + H]^+^. The NMR spectra of **3** displayed signals of a pyrocatechol group [22], a methyl group (δ_H_ 2.49 s; δ_C_ 23.2 t) (Table 2), and a 1,4-disubstituted pyridine ring. The pyrocatechol group was connected to C-5 in the pyridine ring according to HMBC correlation from H-4, H-6 to C-1′, and H-2′, H-6′ to C-5. The methyl group was placed at C-2 on the basis of HMBC correlation from H-7 to C-2 (Figure 3). Therefore, the structure of compound **3** was determined as rynchopeterine C, whose assigned NMR data were listed in Table 2.

**Figure 4:**
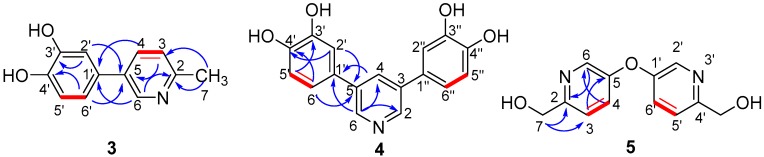
Key ^1^H-^1^H COSY (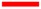) and HMBC (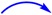) correlations of compounds **3**–**5**.

Compound **4** was isolated as a light yellow powder, and its molecular formula was deduced to be C_17_H_13_NO_4_ with an unsaturation degree of twelve by HR-ESIMS at *m*/*z*: 296.0920 [M + H]^+^. Full analysis of the MS and NMR spectra of **4** demonstrated that it is composed of two pyrocatechol groups and a 3,5-disubstituted pyridine ring. The connection of those three fragments was established by the HMBC correlations from H-2′ and H-6′ to C-5, and H-4 and H-6 to C-1′ (Figure 4). Hence, the structure of compound **4** was determined, with its assigned NMR data listed in Table 2.

Compound **5** was isolated as a white powder, whose molecular formula was deduced to be C_12_H_12_N_2_O_3_ with an unsaturation degree of eight by HR-ESIMS at *m*/*z*: 233.0920 [M + H]^+^. The NMR spectra of **5** displayed signals of a 2,5-disubstituted pyridine ring, an oxygenated methylene. The oxygenated methylene was placed at C-2 on the basis of HMBC correlation from H-7 to C-2 (Figure 4). In combination with the comparison of the NMR data of **5** with those of 5-hydroxy-2-pyridinemethanol, the MS data indicated that **5** is composed of two identical fragments. Thus, the structure of compound **5** was determined, with its assigned NMR data listed in Table 2.

### 2.2. Antioxidant and Anti-Tumor Activities

Our previous studies have found that the ethyl acetate fraction of *B. rynchopetera* exhibited strong 1,1-diphenyl-2-picrylhydrazyl (DPPH) radical scavenging activity, with an IC_50_ value of 23.35 μg/mL, while the IC_50_ values of petroleum ether (PE) and n-butyl alcohol fractions were 673.0 and 620.3 μg/mL, respectively. The results indicated that the ethyl acetate fraction is abundant in antioxidant ingredients, which inspired us to measure the anti-radical activity of the isolated compounds. As shown in Table 3, some of the isolated compounds showed impressive anti-radical activity. Especially, compound **7** exhibited significant anti-radical activity with an IC_50_ value of 1.02 μg/mL. The results revealed that new compounds **1**–**4** were nearly equally effective as the positive controls vitamin C and rutin regarding DPPH radical-scavenging activity. Their effective anti-radical activities could be attributed to the hydroxyl group in the molecules [23]. 

The cytotoxicity of compounds **1**–**5** was tested in vitro against ten common human tumor cell lines A549, AGS, Caco-2, Hela, HepG2, K562, MDA-MB-231, TE-1, U251, and Bel-7402 using the modified 3-(4,5-dimethyl-2-thiazolyl)-2,5-diphenyl-2-H-tetrazolium bromide (MTT) method. The results showed that compound **2** exhibited anti-tumor activity against Caco-2 cell and A549 cells with IC_50_ values of 119.7 μg/mL and 108.5 μg/mL, respectively. Compound **3** exhibited mild anti-tumor activity against Caco-2 cells with an IC_50_ value of 158.7 μg/mL, while the positive control cisplatin generated IC_50_ values of 6.2 μg/mL against Caco-2 cells.

Phenolics have been reported to exhibit various physiological effects in humans, including inhibiting platelet aggregation, reducing the risk of coronary heart disease and cancer, and preventing oxidative damage to lipids and low-density lipoproteins; these effects are generally associated with their antioxidant abilities [24]. The antioxidant phenolics in *B. rynchopetera* might be responsible for its therapeutic effect. *B. rynchopetera* are utilized to treat various cancer by the natives; the isolated compounds **2** and **3** showed mild anti-tumor activities, and could be regarded as the effective pharmaceutical constituents in *B. rynchopetera*.

## 3. Experimental Section

### 3.1. General Experimental Procedures

NMR spectra were acquired with a Bruker AV-400 spectrometer (Bruker, Karlsruhe, Germany) using TMS as the internal reference. MS analyses were recorded with an Agilent G3250AA LC/MSD TOF System (Agilent, Santa Clara, CA, USA). Silica gel (300–400 mesh; Qingdao Haiyang Chemical Factory, Qingdao, China), Sephadex LH-20 (GE Healthcare, Fairfield, NJ, USA), and ODS (Merck China, Beijing, China) were used for column chromatography (CC). GF254 plates (Qingdao Marine, Qingdao, China) were used for thin layer chromatography, and spots were visualized under UV light or by spraying with 5% H_2_SO_4_ in ethanol followed by heating.

### 3.2. Insect Material

*B. rynchopetera* was collected from Dali Bai Autonomous Prefecture in Yunnan Province of China in June 2008, and identified by Professor Guodong Ren from Hebei University. A voucher specimen (No. 2008071001) is deposited in the special medicinal insect development national engineering research center of medicinal insect specimens of Dali University.

### 3.3. Extraction and Isolation

Air-dried and powdered *B. rynchopetera* (5.0 kg) were extracted by 95% ethanol for three times under the room temperature. Removal of the solvent under reduced pressure afforded the crude extract, which was partitioned successively with PE, ethyl acetate and n-butyl alcohol to yield three soluble fractions.

The ethyl acetate fraction was subjected to silica gel CC eluted with PE–acetone gradient system (10:1 to 0:10) to give eleven fractions (FrA-FrK). Further silica gel CC purification of FrC was accomplished by elution with CHCl_3_-CH_3_OH (100:1 to 3:1) to afford fractions FrC1–FrC7. FrC2 was further subjected to silica gel, ODS, and Sephadex LH-20 CC to provide compounds **10** (30 mg), **11** (18 mg), **12** (10 mg), and **14** (8 mg). FrC3 was further subjected to ODS and silica gel CC to provide compounds **1** (50 mg), **8** (5 mg), **9** (8 mg), **13** (18 mg), and **16** (25 mg). FrC4 was further subjected to ODS and silica gel CC to provide compounds **5** (9 mg), **6** (8 mg), **7** (10 mg), and **15** (7 mg). Further silica gel CC purification of FrC5 was accomplished by elution with ODS, silica gel, and Sephadex LH-20 to afford compounds **2** (5 mg), **3** (8 mg), **15** (28 mg), and **18** (12 mg). FrD was further subjected to silica gel CC eluted with CHCl_3_–CH_3_OH (50:1 to 3:1) to afford compound **5** (30 mg). FrE was further subjected to ODS CC eluted with CH_3_OH-H_2_O gradient system (1:9 to 9:1) to provide compounds **4** (30 mg) and **17** (18 mg).

#### 3.3.1. Rynchopeterine A (**1**)

Light yellow liquid; ${\left[\alpha \right]}_{D}^{20}$ −13.8 (c 0.24, CH_3_OH); HR-ESIMS: *m*/*z* 249.0738 [M + Na]^+^ (calcd. for C_11_H_14_O_5_Na: 249.0739); For ^1^H- and ^13^C-NMR spectral data, see Table 1; IR (KBr, cm^−1^) ν_max_ 3410, 1736, 1607, 1591, 1553, 1490, 1456, 1381, 1250, 1165, 1033, 818.

#### 3.3.2. Rynchopeterine B (**2**)

Light brown liquid; HR-ESIMS: *m*/*z* 413.1192 [M + Na]^+^, (calcd. for C_20_H_22_O_8_Na: 413.1212); For ^1^H- and ^13^C-NMR spectral data, see Table 1; IR (KBr, cm^−1^) ν_max_: 3342, 2959, 1740, 1603, 1519, 1430, 1290, 1195, 1110, 944, 805.

#### 3.3.3. Rynchopeterine C (**3**)

White powder; HR-ESIMS: *m*/*z* 202.0859 [M + H]^+^ (calcd. for C_12_H_12_NO_2_: 202.0868); For ^1^H- and ^13^C-NMR spectral data, see Table 2; IR (KBr, cm^−1^) ν_max_: 3380, 3029, 1603, 1595, 1519, 1479, 1380, 1287, 1184, 1065, 802.

#### 3.3.4. Rynchopeterine D (**4**)

Light yellow powder; HR-ESIMS: *m*/*z* 296.0920 [M + H]^+^ (calcd. for C_17_H_14_NO_4_: 296.0923); For ^1^H- and ^13^C-NMR spectral data, see Table 2; IR (KBr, cm^−1^) ν_max_: 3382, 2901, 1603, 1594, 1519, 1455, 1286, 866.

#### 3.3.5. Rynchopeterine E (**5**)

White powder; HR-ESIMS: *m*/*z* 233.0920 [M + H]^+^ (calcd. for C_12_H_13_N_2_O_3_: 233.0926); For ^1^H- and ^13^C-NMR spectral data, see Table 2; IR (KBr, cm^−1^) ν_max_ 3410, 3025, 1605, 1570, 1520, 1483, 1285, 1069, 1010, 812.

### 3.4. DPPH Assay

The DPPH radical-scavenging activity was assessed using a previously published method [25]. A total of 3.9 mL DPPH (0.075 mM) was mixed with 0.1 mL sample at various concentrations. The mixture was shaken and incubated at 25 °C in the dark, and the absorbance of the reaction mixture was measured at 515 nm after 30 min. The DPPH radical-scavenging activity was calculated by the following equation: inhibition (%) = (1 − A_s_/A_o_) × 100%; where A_o_ is the absorbance of DPPH without any sample, and A_s_ is the absorbance of a sample with DPPH in it. Inhibition was the scavenging effect, and the 50% absorbance reduction (IC_50_) was measured from a curve relating concentration to the absorbance of a sample. Vitamin C and rutin were used as positive controls.

### 3.5. Anti-Tumor Assay

The cytotoxicity of compounds **1**–**5** against ten human cell lines, including lung cancer cell A549, gastric cancer cell AGS, colon cancer cell Caco-2, cervical cancer cell Hela, hepatoma cell HepG2 and Bel-7402, leukemia cell K562, breast cancer cell MDA-MB-231, esophageal carcinoma cell TE-1, and glioma cell U251 were determined in vitro by the MTT method [26]. Briefly, cells were seeded in 96-well plates at a density of 5 × 10^3^ to 1 × 10^4^ cells/well. Cells were treated with different concentrations of compounds and positive control for 48 h, following incubation with MTT solution for 4 h. The absorbance was measured using a microplate reader at 490 nm. Cisplatin was used as a positive control.

## Figures and Tables

**Table 1:** NMR spectral data of compounds **1** (CD_3_COCD_3_) and **2** (CD_3_COCD_3_:DMSO-*d*_6_ = 5:1).

No.	1		No.	2	
	δ_H_ (*J* in Hz)	δ_C_		δ_H_ (*J* in Hz)	δ_C_
1		129.5 s	1 (1″)		129.6 s
2	6.76 (d, 2.0)	116.0 d	2 (2″)	6.76 (2H, d, 1.6)	116.0 d
3		145.0 s	3 (3″)		145.0 s
4		143.7 s	4 (4″)		143.7 s
5	6.76 (d, 8.0)	115.2 d	5 (5″)	6.76 (2H, d, 8.0)	115.3 d
6	6.59 (dd, 8.0, 2.0)	120.2 d	6 (6″)	6.59 (2H, dd, 8.0, 1.6)	120.2 d
7	2.80 (2H, t, 7.0)	34.2 t	7 (7″)	2.78 (4H, t, 7.0)	34.2 t
8	4.21 (dt, 7.0, 11.6)	65.3 t	8 (8″)	4.20 (4H, t, 7.0)	65.2 t
	4.30 (dt, 7.0, 11.6)		1′ (4′)		171.8 s
1′		174.7 s	2′ (3′)	2.59 (4H, s)	28.5 t
2′	4.23 (q, 7.0)	66.6 d			
3′	1.31 (3H, d, 7.0)	19.9 q			
OH-3	7.80 (brs)				
OH-4	7.77 (brs)				

**Table 2:** NMR spectral data of compounds **3**–**5** (CD_3_OD).

No.	3		No.	4		No.	5	
	δ_H_ (*J* in Hz)	δ_C_		δ_H_ (*J* in Hz)	δ_C_		δ_H_ (*J* in Hz)	δ_C_
1			1			1 (3′)		
2		156.0 s	2, 6	8.59 (brs)	144.2 d	2 (4′)		152.1 s
3	7.24 (d, 8.0)	123.2 d	3, 5		137.3 s	3 (5′)	7.34 (d, 8.4)	120.9 d
4	7.80 (dd, 8.0, 2.2)	133.7 d	4	8.06 (t, 2.0)	131.9 d	4 (6′)	7.25 (dd, 8.4, 2.8)	122.9 d
5		133.6 s	1′ (1″)		128.9 s	5 (1′)		152.5 s
6	8.64 (d, 2.2)	146.7 d	2′ (2″)	7.14 (d, 2.2)	113.6 d	6 (2′)	8.15 (d, 2.8)	136.3 d
7	2.49 (3H, s)	23.2 q	3′ (3″)		145.7 s	7 (7′)	4.61 (s)	64.1 t
1′		129.3 s	4′ (4″)		145.9 s			
2′	7.11 (d, 2.2)	113.9 d	5′ (5″)	6.92 (d, 8.2)	115.7 d			
3′		146.1 s	6′ (6″)	7.06 (dd, 8.2, 2.2)	118.4 d			
4′		145.8 s						
5′	6.90 (d, 8.1)	116.1 d						
6′	6.97 (dd, 8.1, 2.2)	117.9 d						

**Table 3:** DPPH radical-scavenging activity of the compounds from *B. rynchopetera* (IC_50_, μg/mL).

Compound	IC_50_	Compound	IC_50_
**1**	9.02	**15**	5.85
**2**	12.31	**16**	9.09
**3**	7.67	**18**	11.81
**4**	11.86	vitamin C	6.92
**7**	1.02	rutin	8.28

## Supplementary Materials

Supplementary Materials are available online.
