# Supplementary material for: Five New Phenolic Compounds with Antioxidant Activities from the Medicinal Insect Blaps rynchopetera
Source: Molecules. 2017 Aug 4;22(8):1301. doi: 10.3390/molecules22081301 (PMC6152337; doi:10.3390/molecules22081301)
Supplement: Supplementary file 1 [file molecules-22-01301-s001.pdf]

Electronic Supporting Information

Fig. S1. The structures of compounds 6-18.

Fig. S2.^{1}H NMR spectrum of 1 in CD_3COCD_3.

Fig. S3.^{13}C NMR spectrum of 1 in CD_3COCD_3.

Fig. S4.H-H COSY spectrum of 1 in CD_3COCD_3.

Fig. S5.HSQC spectrum of 1 in CD_3COCD_3.

Fig. S6.HMBC spectrum of 1 in CD_3COCD_3.

Fig. S7. HR-ESI-MS spectrum of 1

Fig. S8.^{1}H NMR spectrum of 2 in CD_3COCD_3.

Fig. S9.^{13}C NMR spectrum of 2 in CD_3COCD_3.

Fig. S10. ^{1}H-^{1}H COSY spectrum of 2 in CD_3COCD_3.

Fig. S11.HSQC spectrum of 2 in CD_3COCD_3

Fig. S12.HMBC spectrum of 2 in CD_3COCD_3.

Fig. S13.HR-ESI-MS spectrum of 2.

Fig. S14.^{1}H NMR spectrum of 3 in CD_3COCD_3: DMSO-*d*6 5:1.

Fig. S15.^{13}C NMR spectrum of 3 in CD_3COCD_3: DMSO-*d*6 5:1.

Fig. S16. ^{1}H-^{1}H COSY spectrum of 3 in CD_3COCD_3: DMSO-*d*6 5:1.

Fig. S17. HMQC spectrum of 3 in CD_3COCD_3: DMSO-*d*6 5:1.

Fig. S18. HMBC spectrum of 3 in CD_3COCD_3: DMSO-*d*6 5:1.

Fig. S19. HR-ESI-MS spectrum of 3.

Fig. S20. ^{1}H NMR spectrum of 4 in CD_3OD.

Fig. S21.^{13}C NMR spectrum of 4 in CD_3OD.

Fig. S22. ^{1}H-^{1}H COSY spectrum of 3 in CD_3OD.

Fig. S23. HMQC spectrum of 4 in CD_3OD.

Fig. S24. HMBC spectrum of 4 in CD_3OD.

Fig. S25.HR-ESI-MS spectrum of 4

Fig. S26.^{1}H NMR spectrum of 5 in CD_3COCD_3.

Fig. S27.^{13}C NMR spectrum of 5 in CD_3COCD_3.

Fig. S28. ^{1}H-^{1}H COSY spectrum of 5 in CD_3COCD_3.

Fig. S29. HMQC spectrum of 5 in CD_3COCD_3.

Fig. S30. HMBC spectrum of 5 in CD_3COCD_3.

Fig. S31. HR-ESI-MS spectrum of 5.

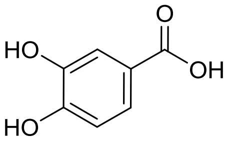

6

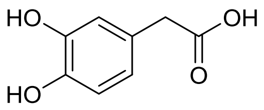

7

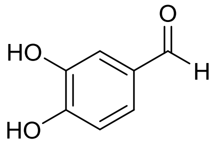

8

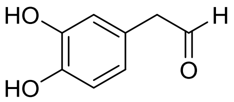

9

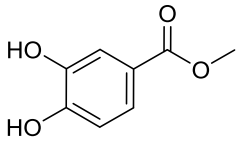

10

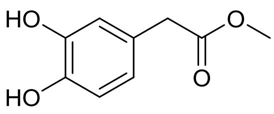

11

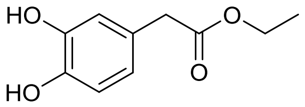

12

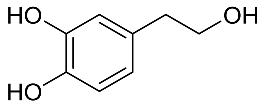

13

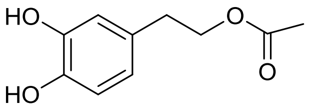

14

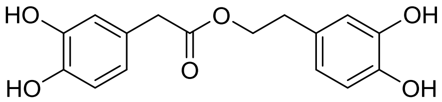

15

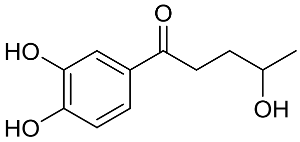

17

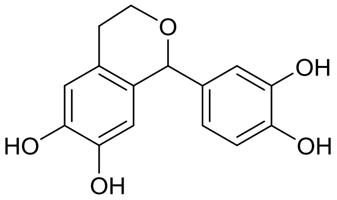

16

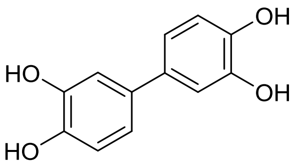

18

Fig. S1. The structures of compounds 6-18.

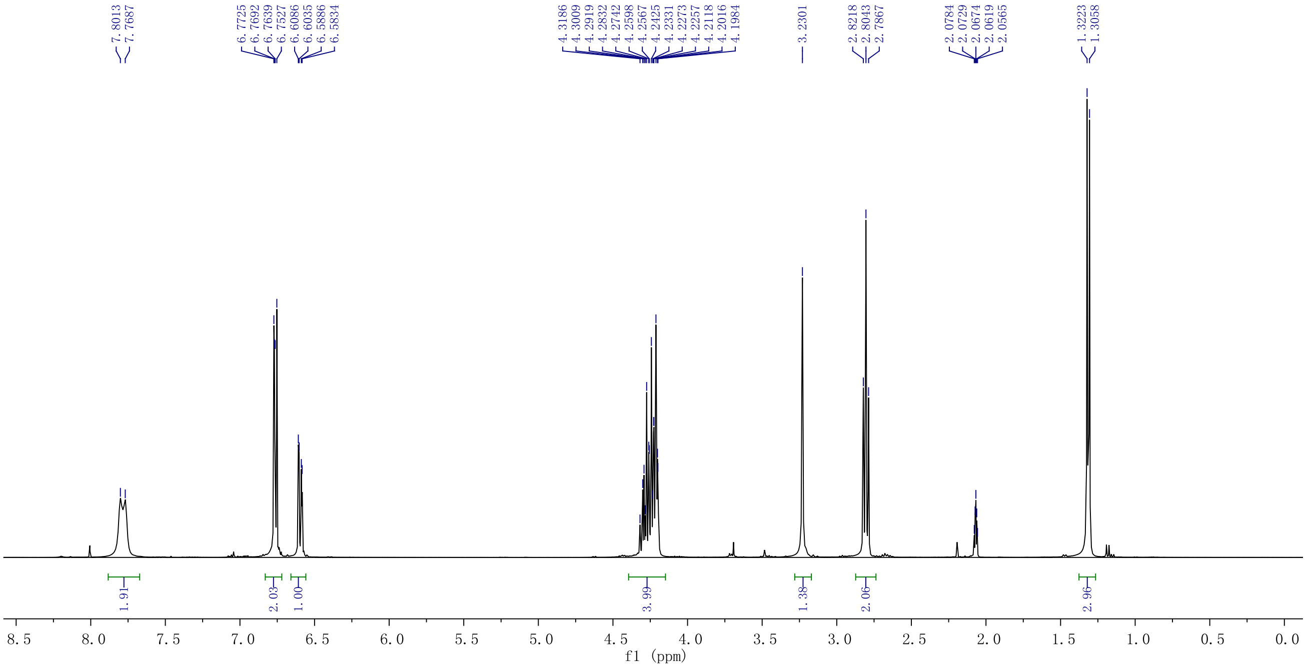

Fig. S2. ^{1}H NMR spectrum of 1 in CD_3COCD_3

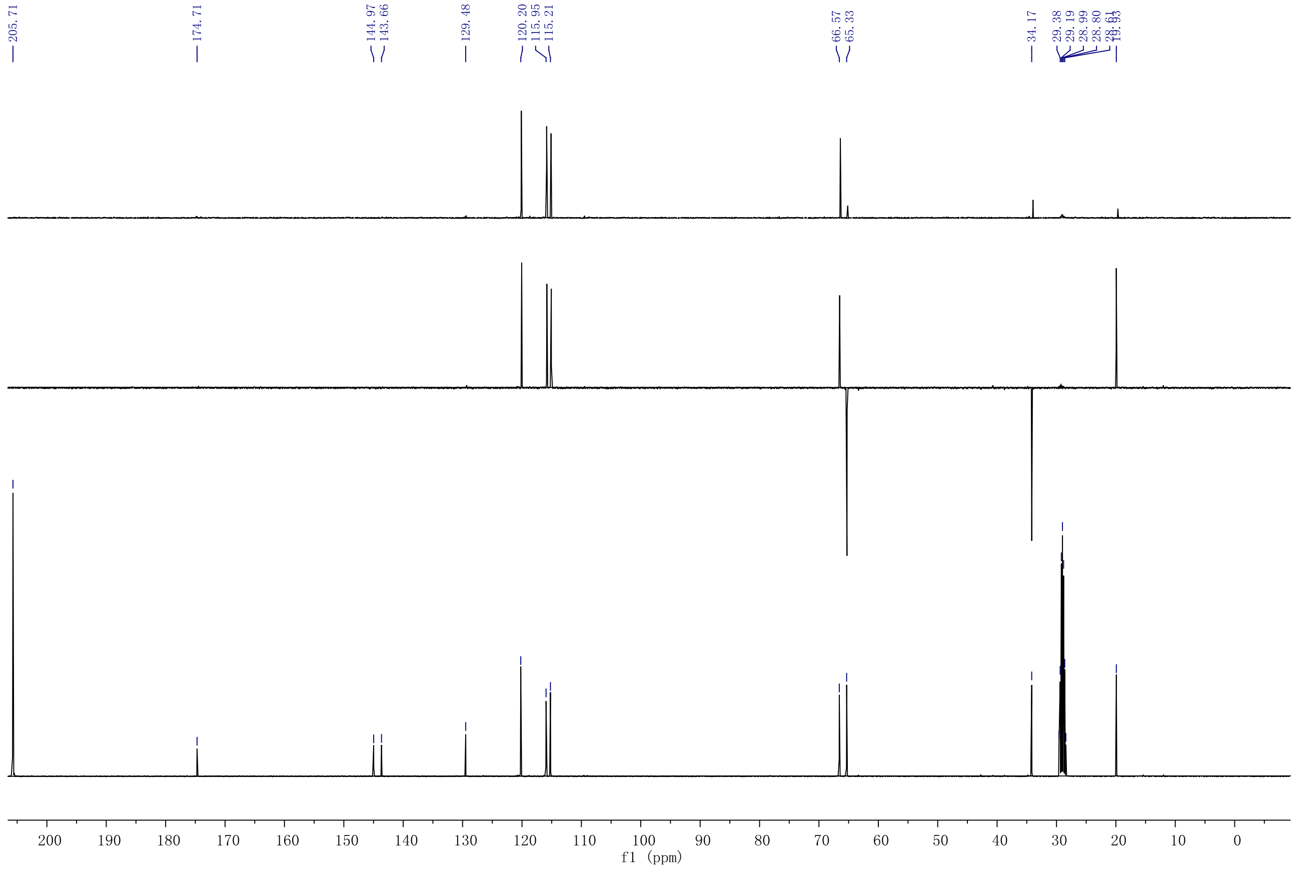

Fig. S3. ^{13}C NMR spectrum of 1 in CD_3COCD_3

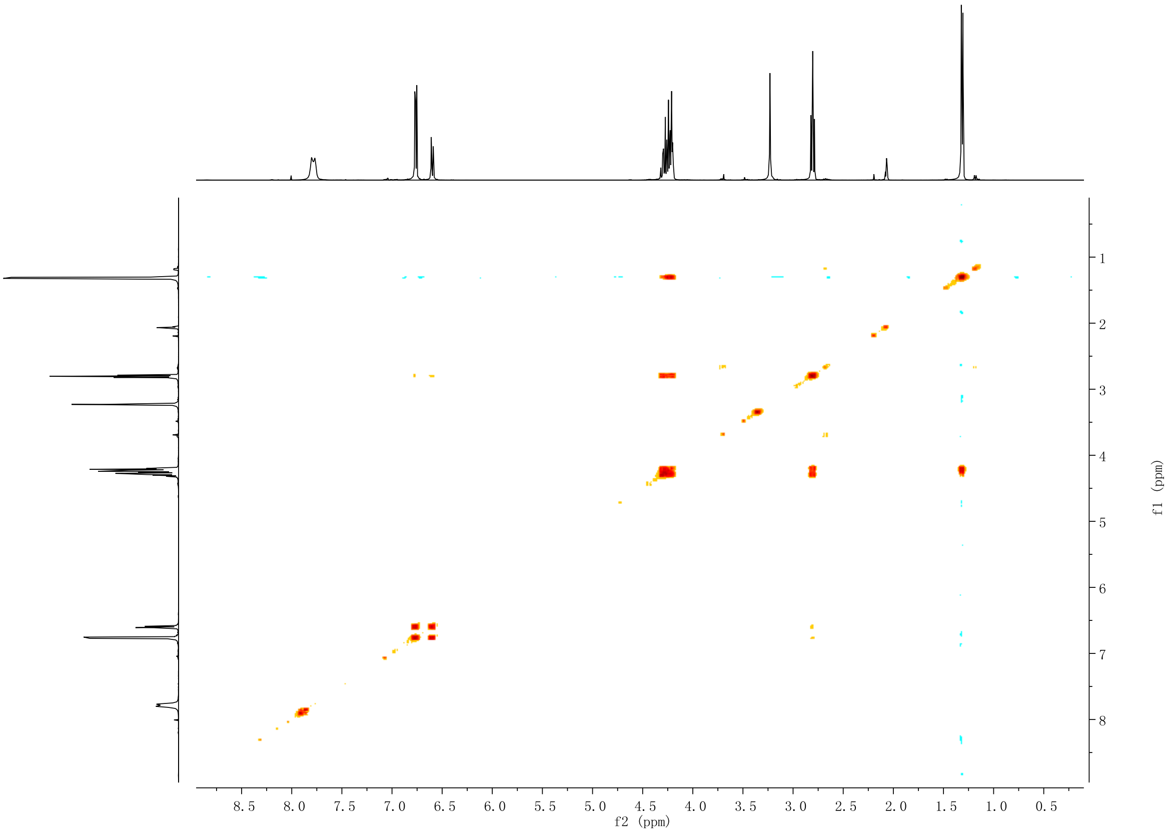

Fig. S4. H-H COSY spectrum of 1 in CD_3COCD_3

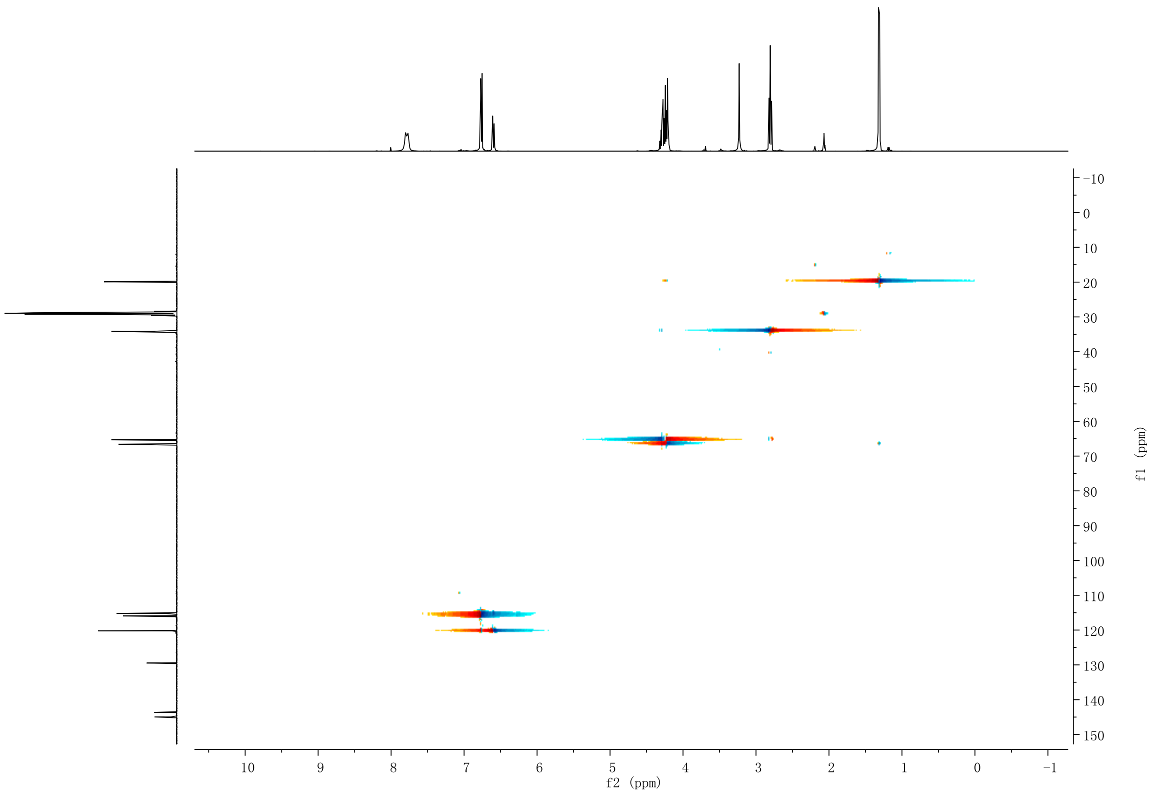

Fig. S5. HSQC spectrum of 1 in CD_3COCD_3

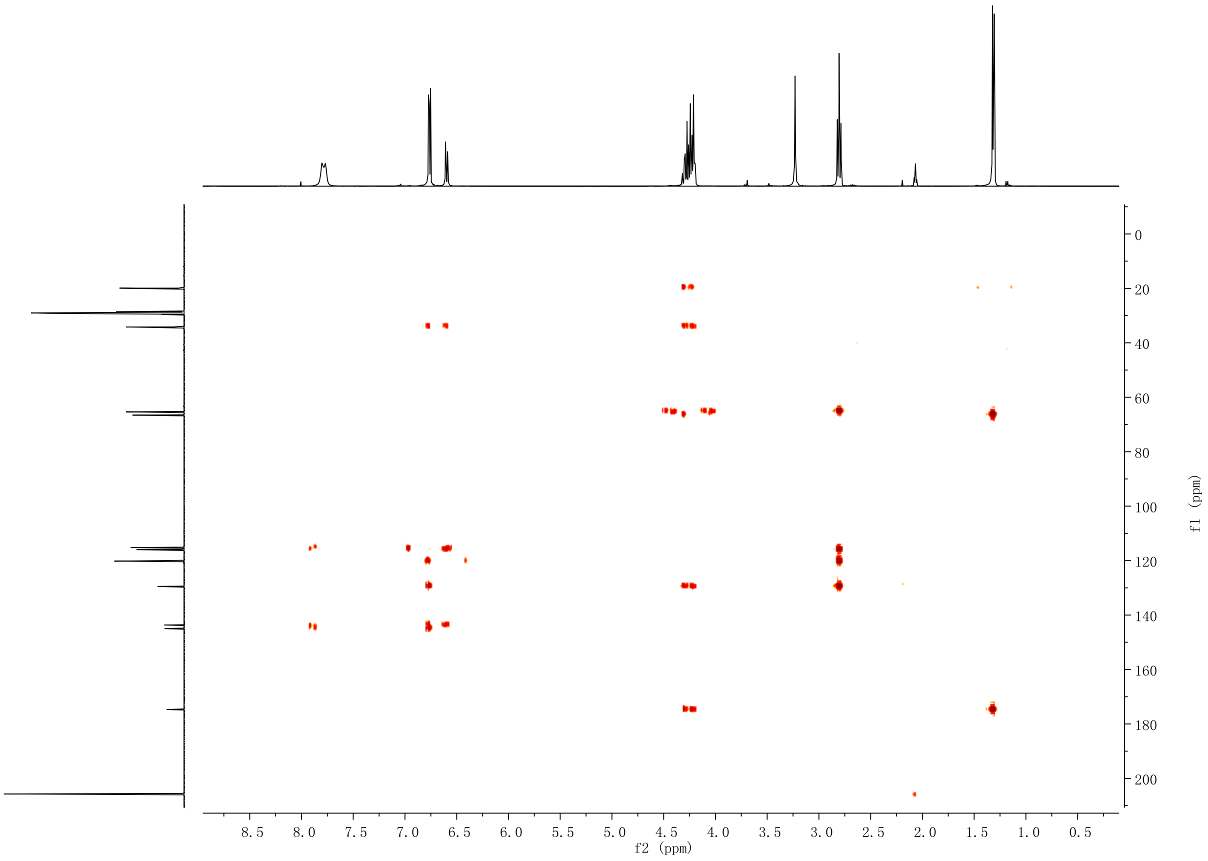

Fig. S6. HMBC spectrum of 1 in CD_3COCD_3

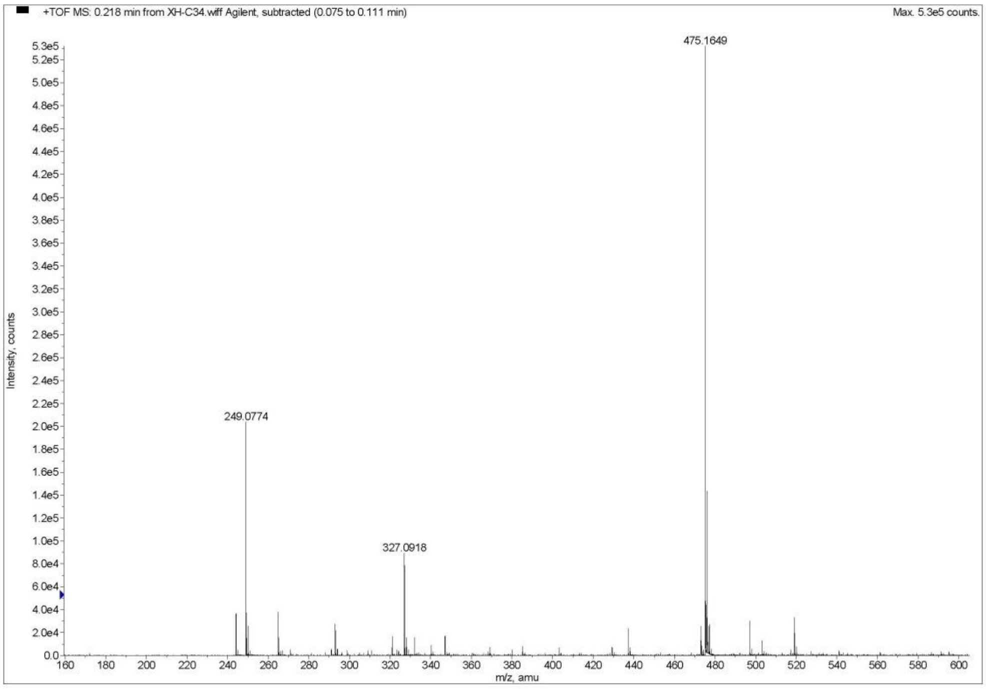

Fig. S7. HR-ESI-MS spectrum of 1.

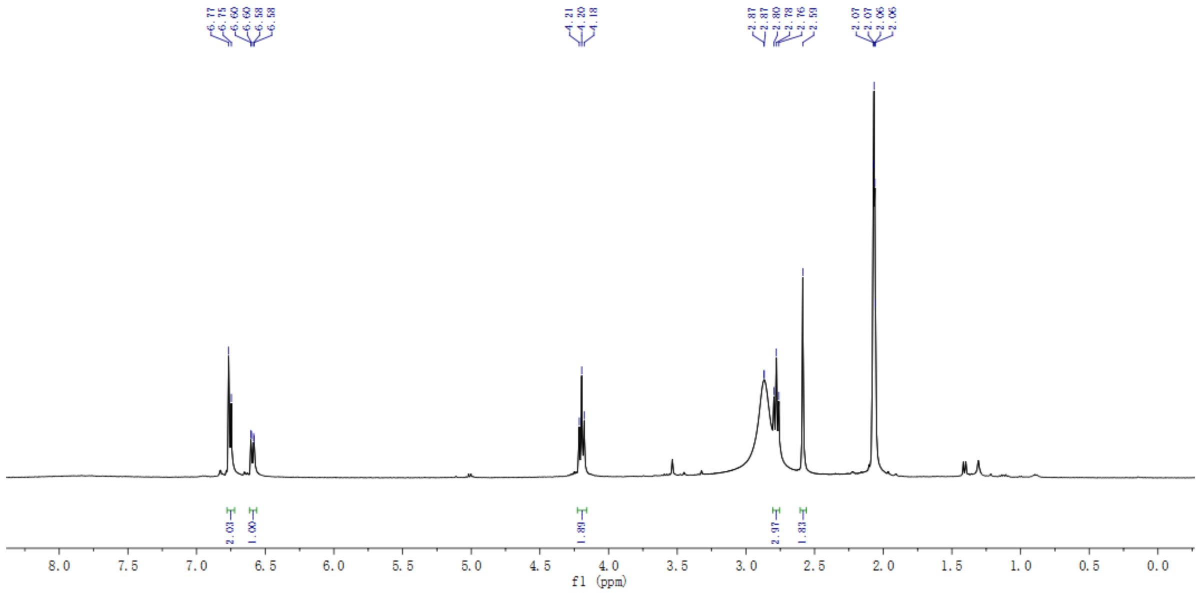

Fig. S8. ^{1}H NMR spectrum of 2 in CD_3COCD_3

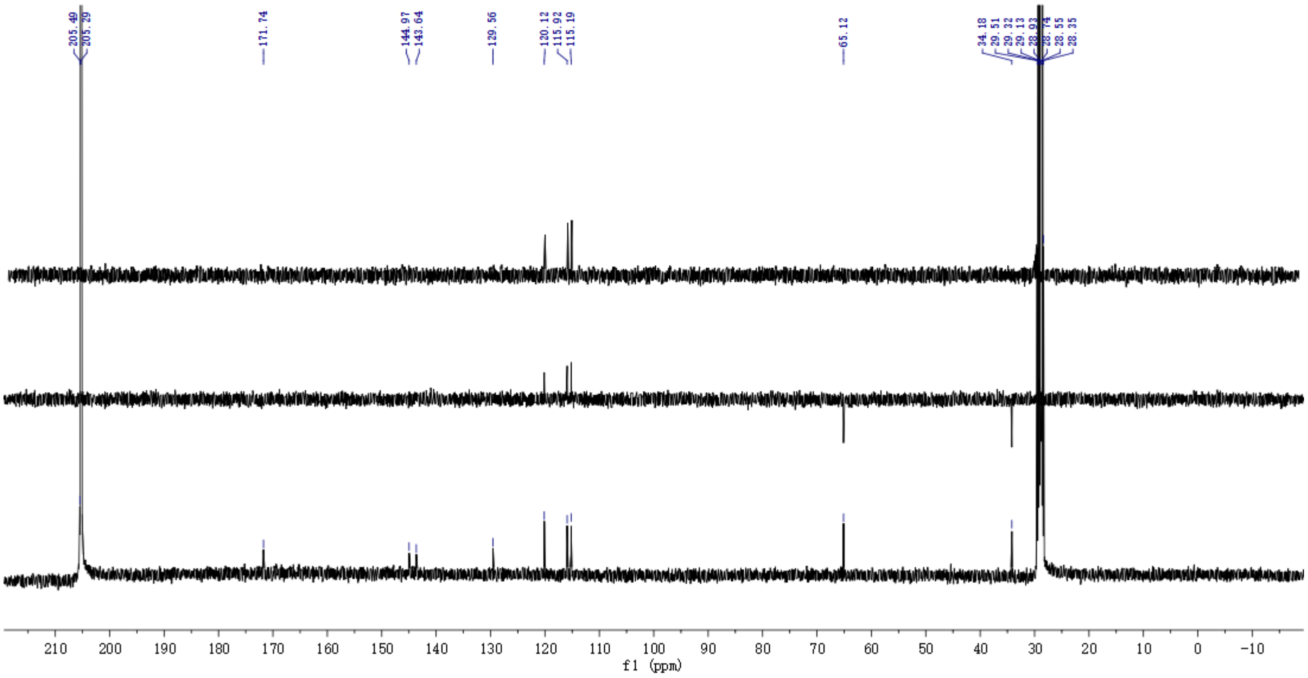

Fig. S9. ^{13}C NMR spectrum of 2 in CD_3COCD_3

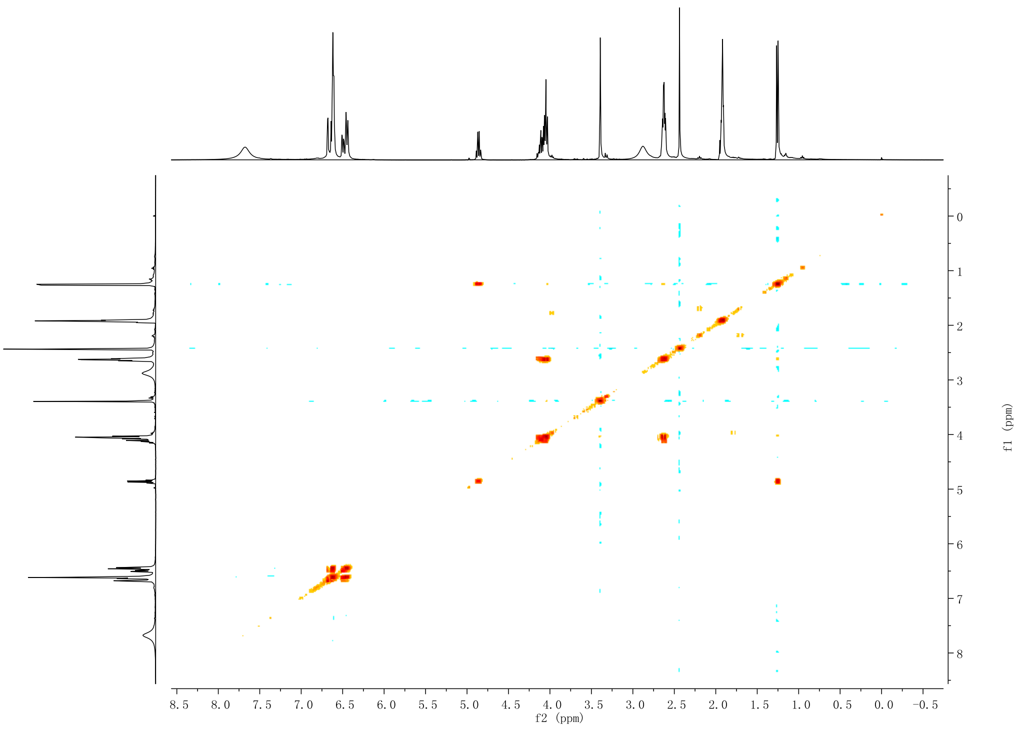

Fig. S10. ^{1}H-^{1}H COSY spectrum of 2 in CD_3COCD_3

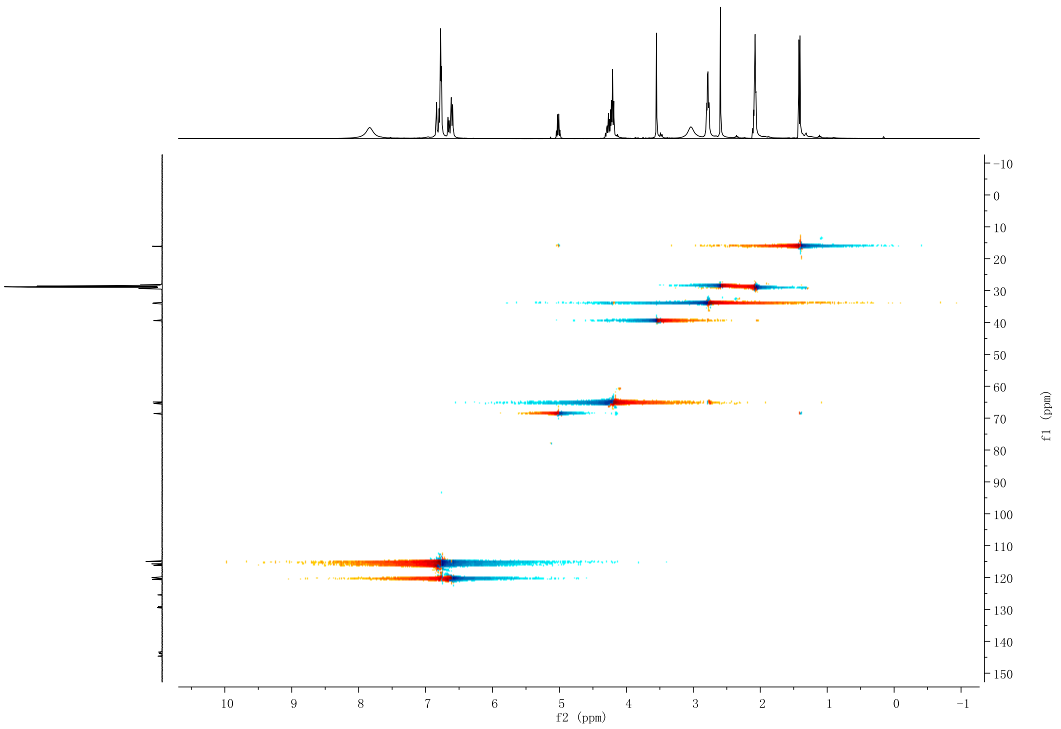

Fig. S11. HSQC spectrum of 2 in CD_3COCD_3

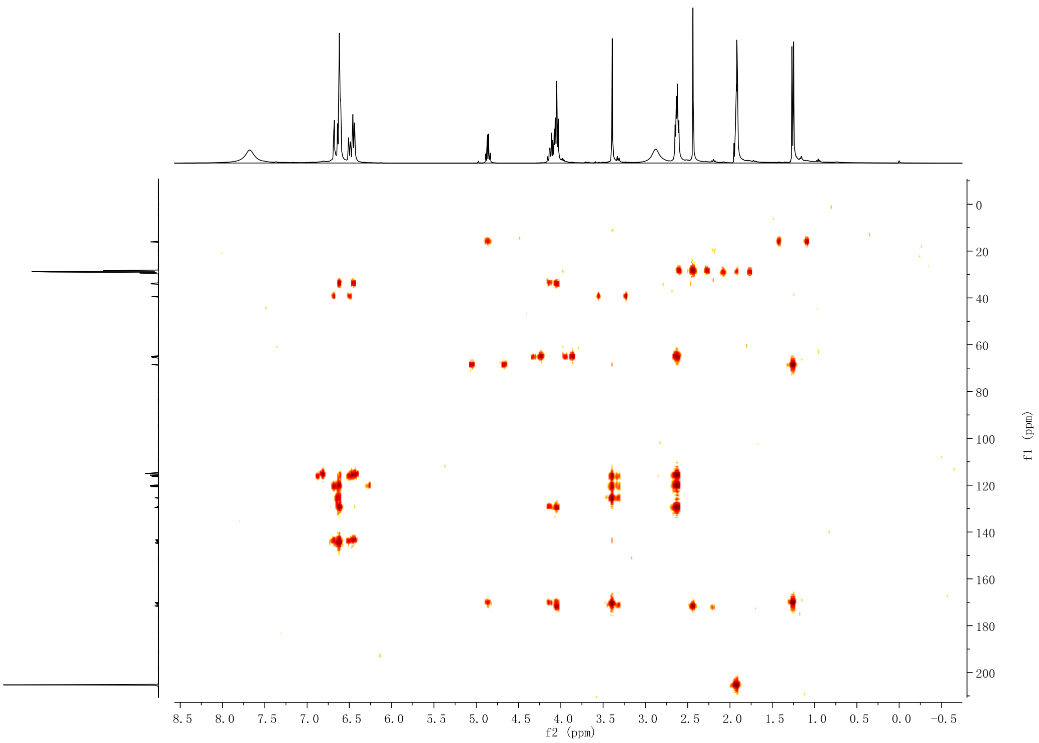

Fig. S12. HMBC spectrum of 2 in CD_3COCD_3

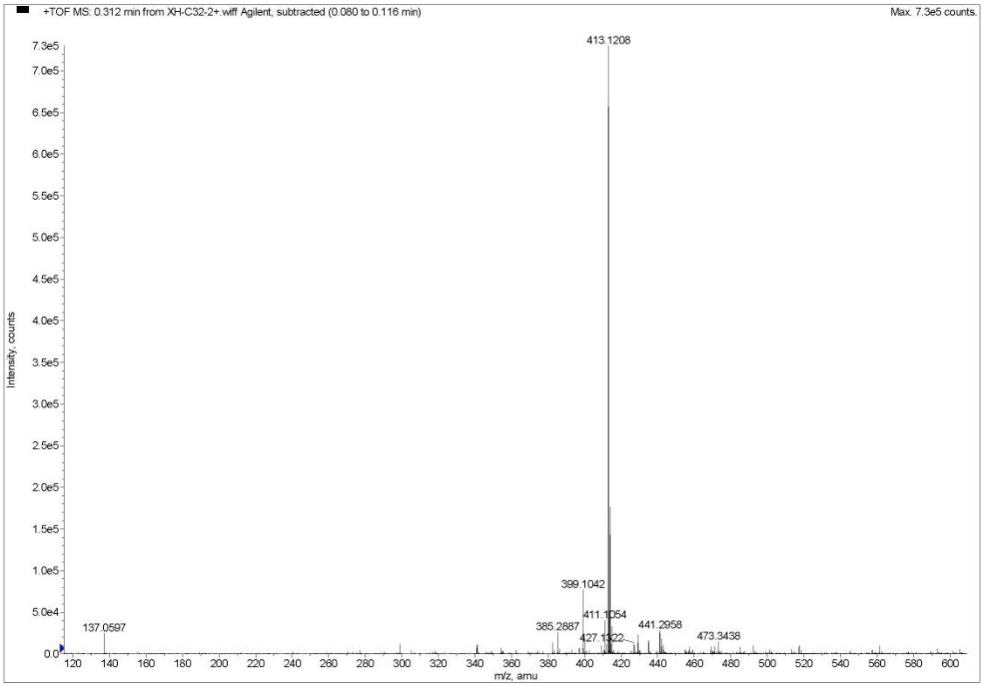

Fig. S13. HR-ESI-MS spectrum of 2.

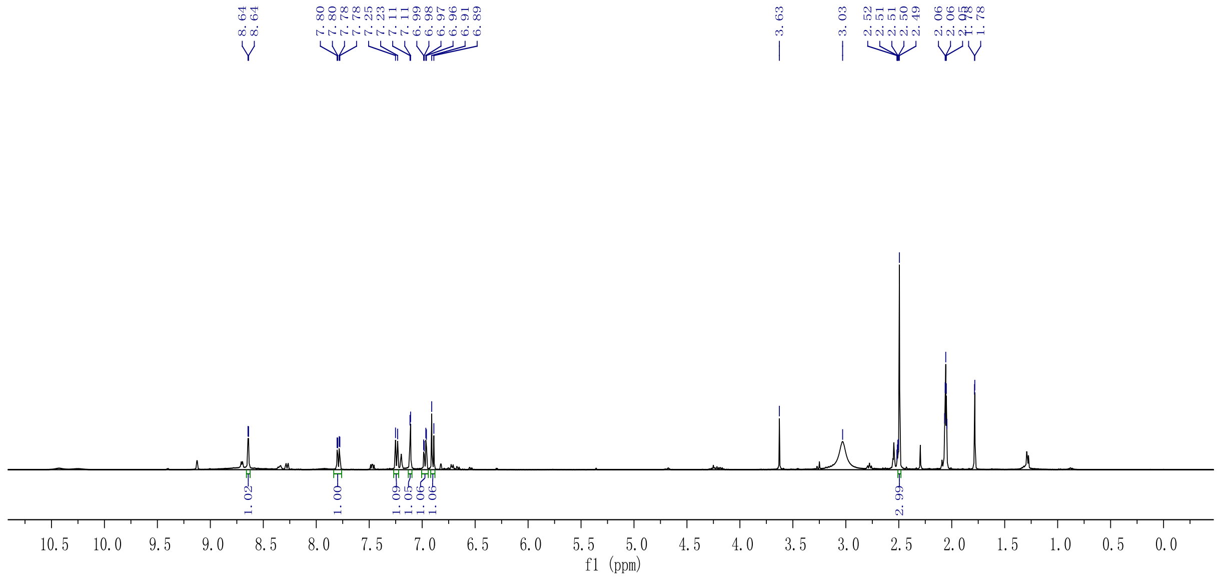

Fig. S14. ^{1}**H NMR spectrum of 3 in** CD_3COCD_3**: DMSO-*d*$_6$ = 5:1**

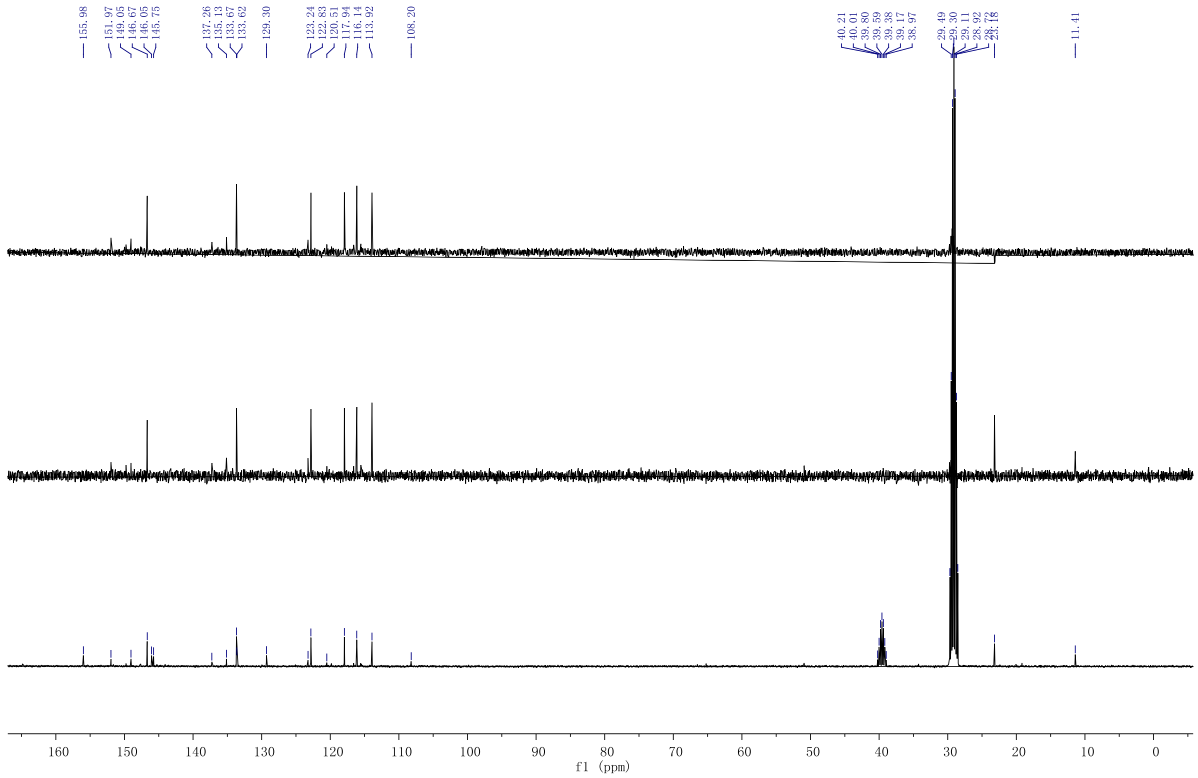

Fig. S15. ^{13}**C NMR spectrum of 3 in** CD_3COCD_3**: DMSO- *d*$_6$ = 5:1**

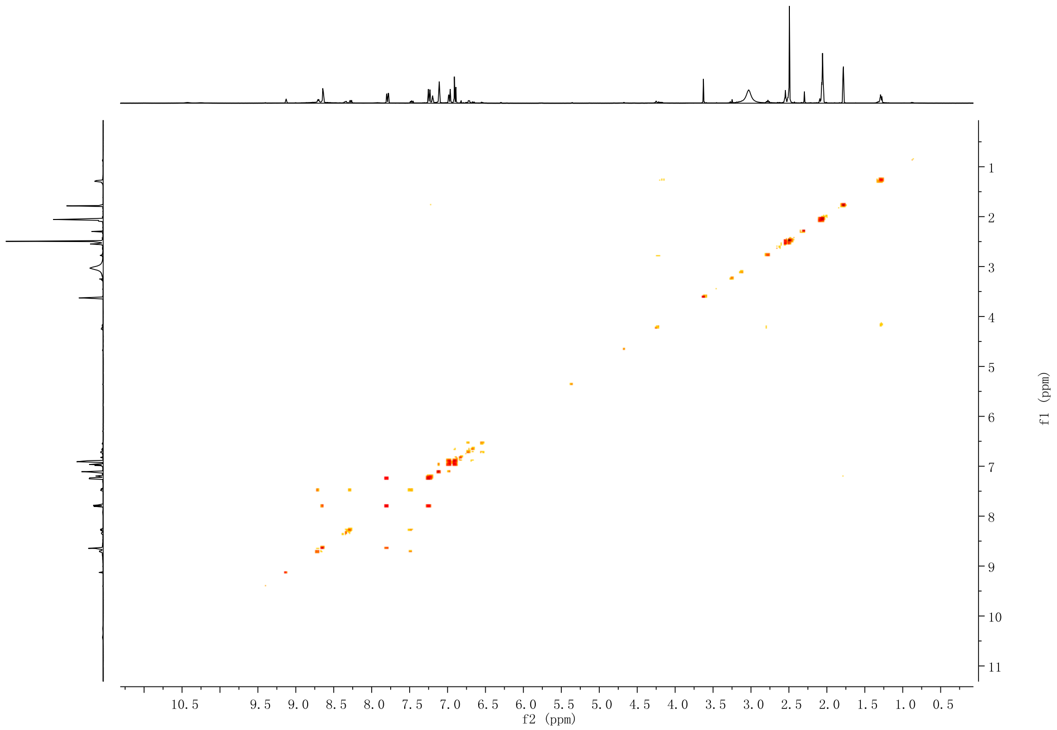

Fig. S16. ^1H-^1H **COSY spectrum of 3 in** CD_3COCD_3**: DMSO-** d_6 **= 5:1**

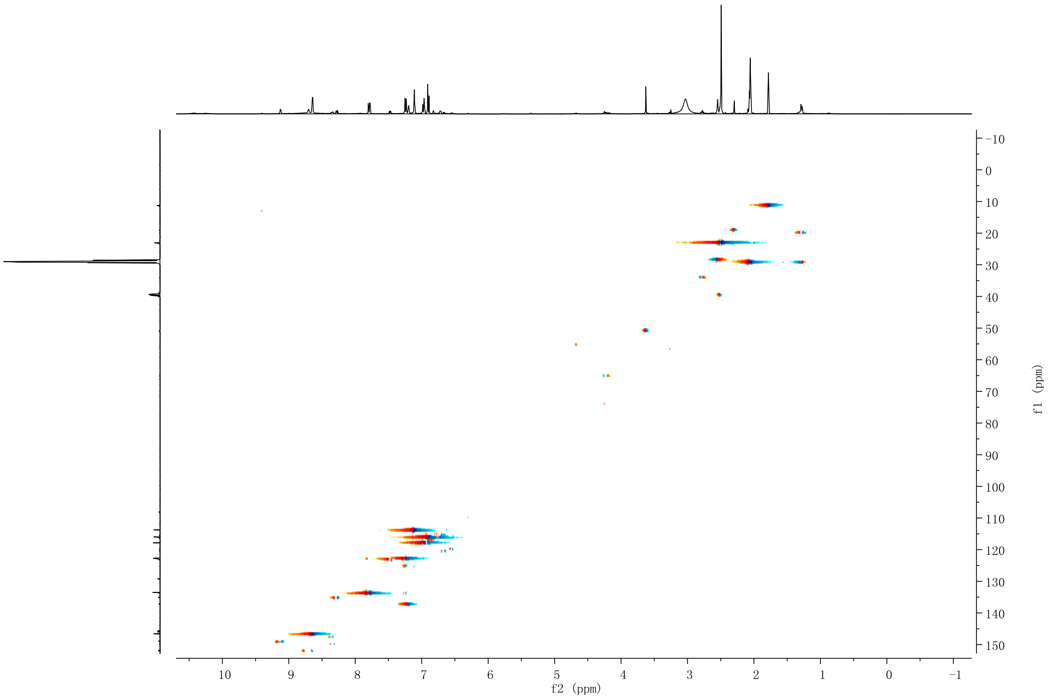

Fig. S17. HSQC spectrum of 3 in CD_3COCD_3**: DMSO-** d_6 **= 5:1**

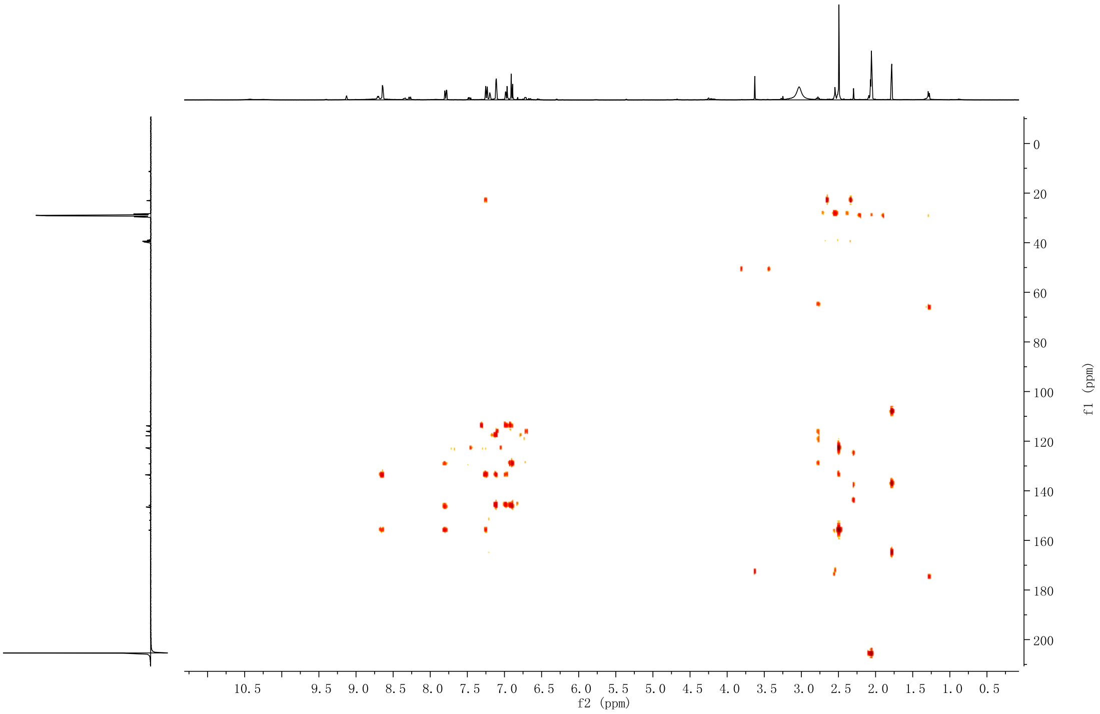

Fig. S18. HMBC spectrum of 3 in CD_3COCD_3: DMSO-d_6 = 5:1

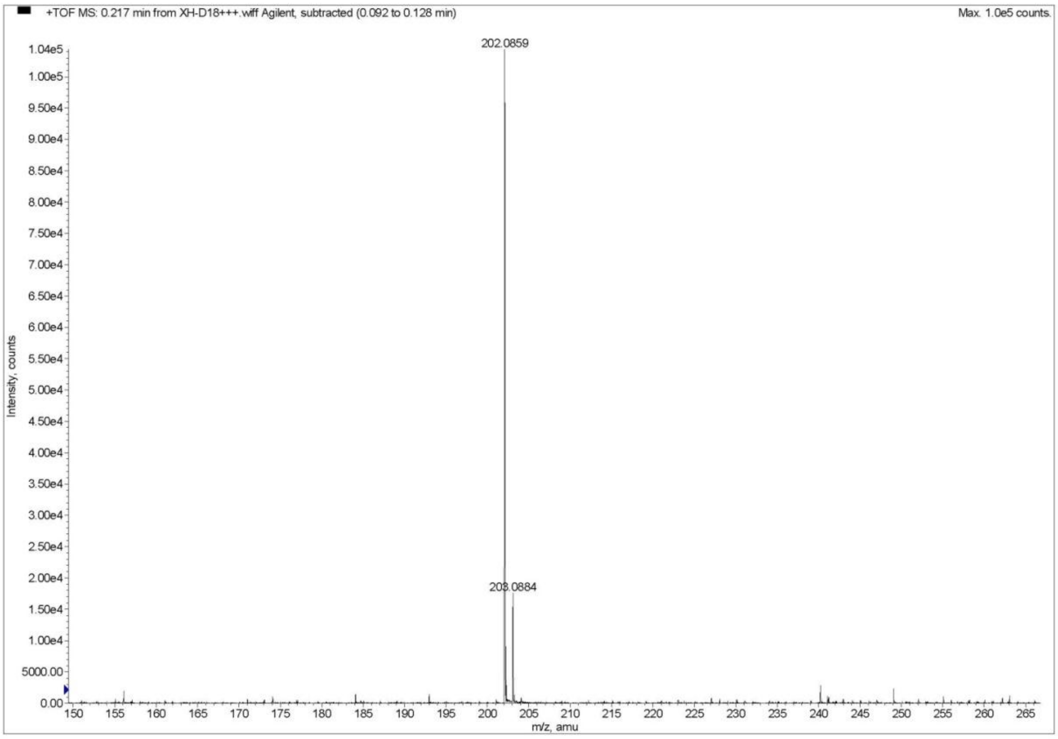

Fig. S19. HR-ESI-MS spectrum of 3

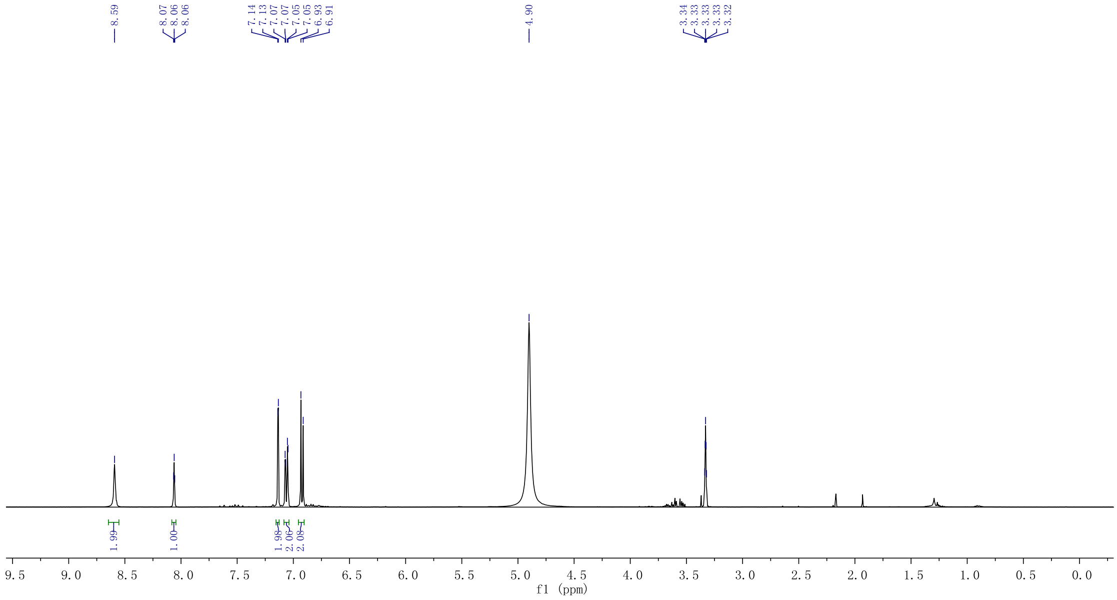

Fig. S20. ¹H-NMR spectrum of 4 in CD_3OD

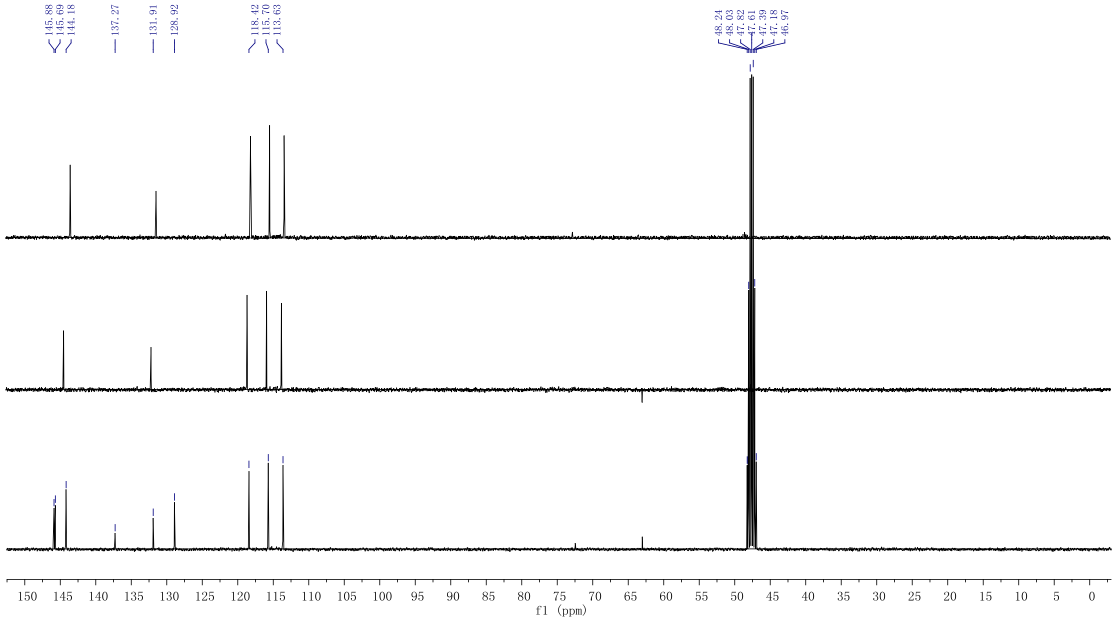

Fig. S21. ¹³C NMR spectrum of 4 in CD_3OD

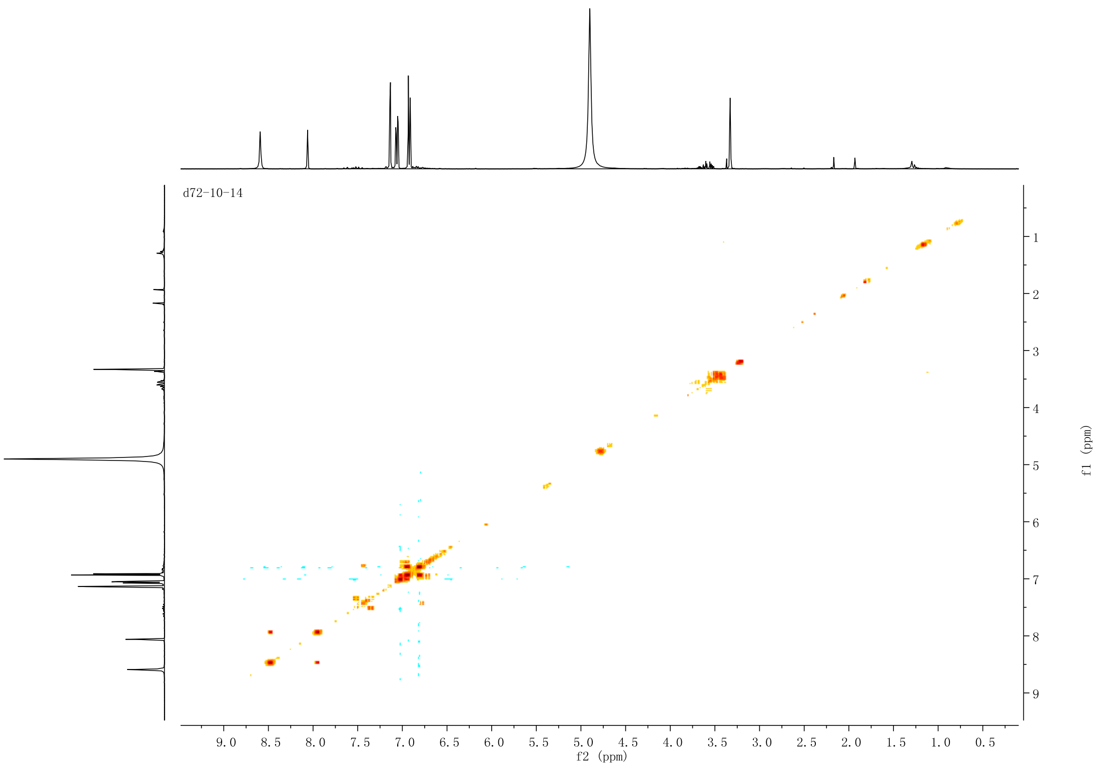

Fig. S22. ^{1}H-^{1}H COSY spectrum of 4 in CD_3OD

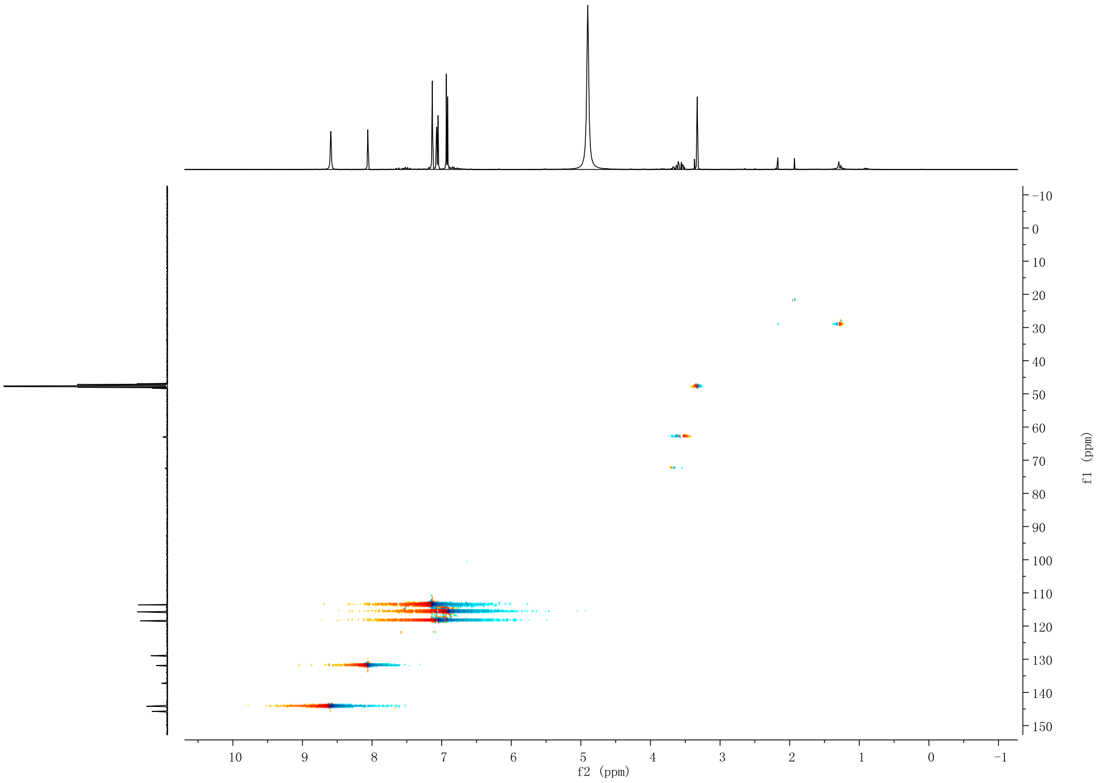

Fig. S23. HSQC spectrum of 4 in CD_3OD

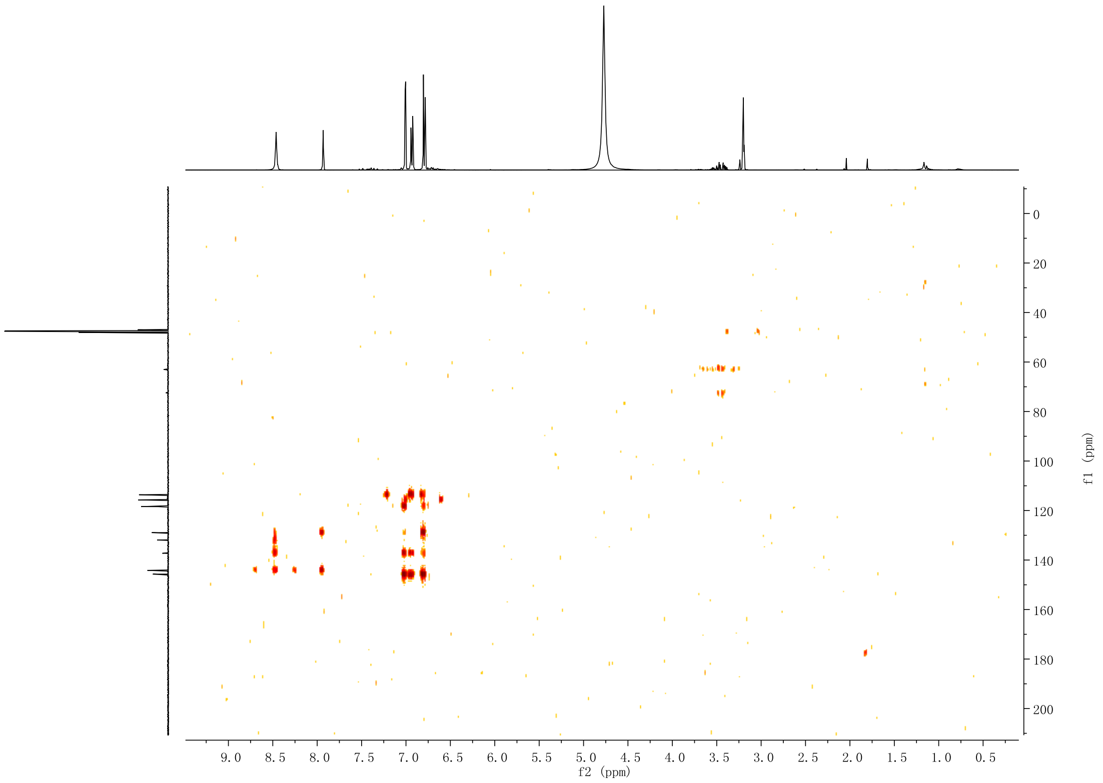

Fig. S24. HMBC spectrum of 4 in CD_3OD

Qualitative Analysis Report

**Data Filename**	XH-D72.d	**Sample Name**	XH-D72
**Sample Type**	Sample	**Position**	P1-D3
**Instrument Name**	Instrument 1	**User Name**	
**Acq Method**	SIBU.m	**Acquired Time**	1/6/2015 1:49:26 PM
**IRM Calibration Status**	Success	**DA Method**	Default.m
**Comment**			

Sample Group **Info.**
Acquisition SW Version 6200 series TOF/6500 series Q-TOF B.05.01 (B5125.2)

User Spectra

Fragmentor Voltage	Collision Energy	Ionization Mode
135	0	ESI

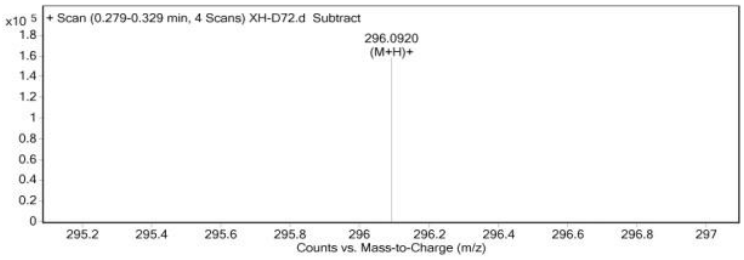

Peak List

m/z	z	Abund	Formula	Ion
296.092	1	158063.61	C17 H13 N O4	(M+H)+
297.0952	1	31812.14	C17 H13 N O4	(M+H)+
297.2396	1	3672.2		
298.0969	1	3902.19	C17 H13 N O4	(M+H)+
301.1419	1	7029.75		
301.2852	1	8580.91		
310.1078	1	5058.93		
341.2665	1	6015.52		
385.2931	1	7487.52		
429.3182	1	4323.08		

Formula Calculator Element Limits

Element	Min	Max
C	3	60
H	0	120
O	0	10
N	0	5

Formula Calculator Results

Formula	CalculatedMass	CalculatedMz	Mz	Diff. (mDa)	Diff. (ppm)	DBE
C17 H13 N O4	295.0845	296.0917	296.0920	-0.3	-1.0	12.0000

--- End Of Report ---

Fig. S25. HR-ESI-MS spectrum of 4

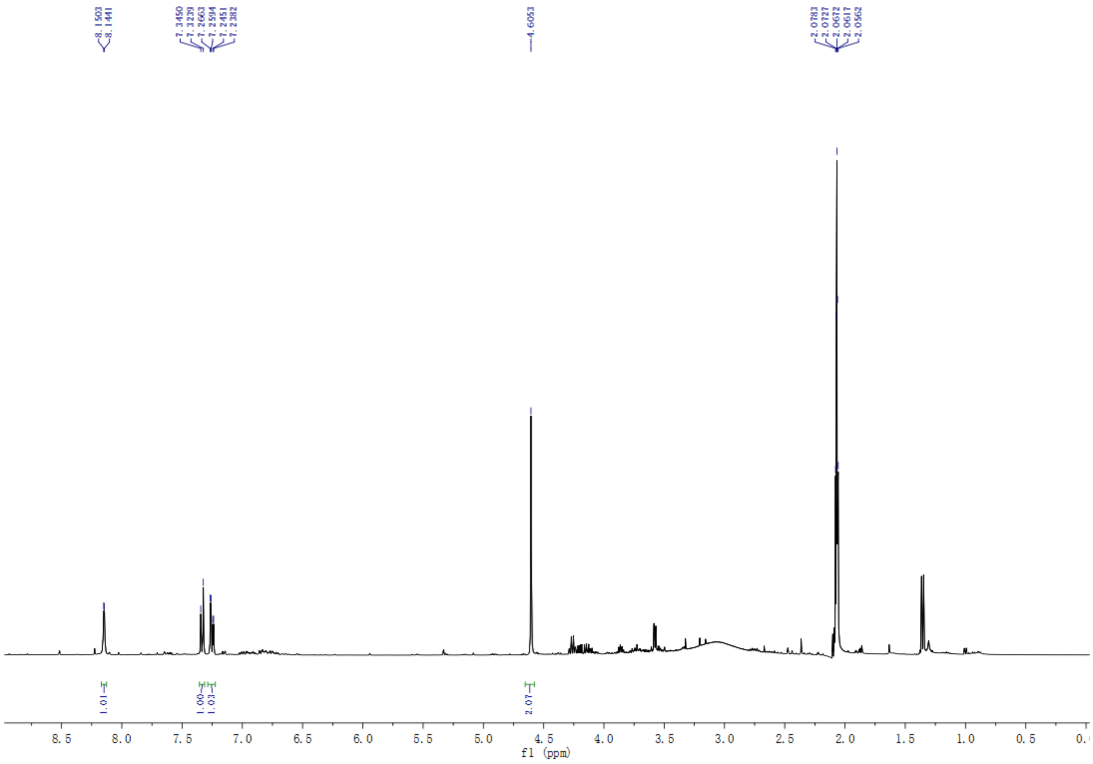

Fig. S26 ^{1}H-NMR spectrum of 5 in CD_3COCD_3

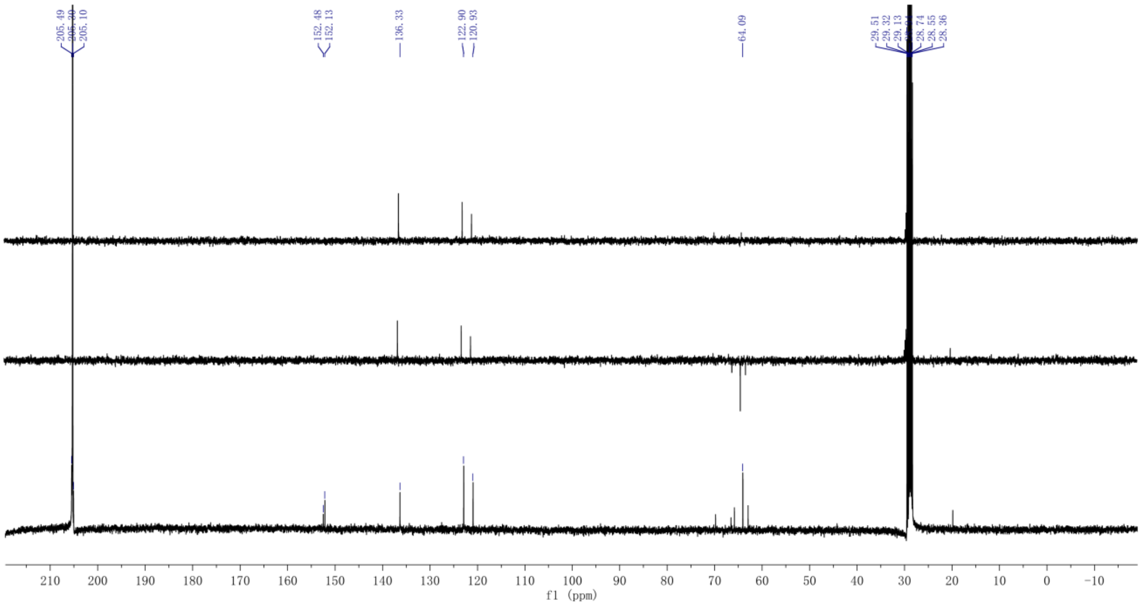

Fig. S27. ^{13}C-NMR spectrum of 5 in CD_3COCD_3

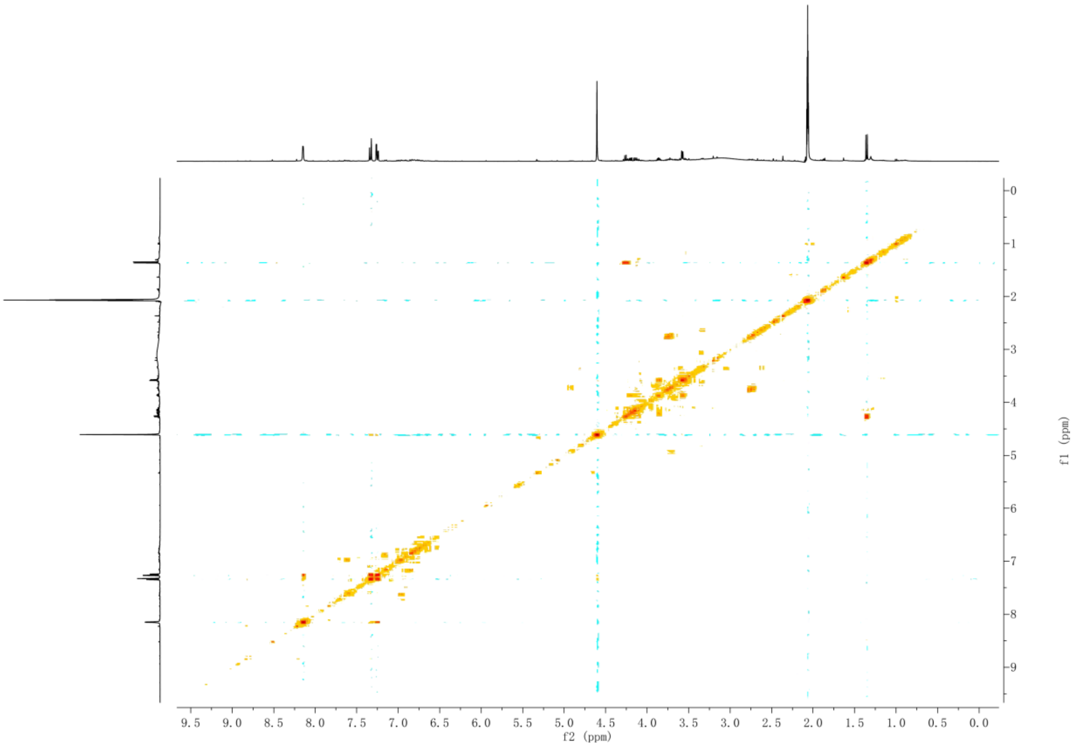

Fig. S28. ^1H-^1H COSY spectrum of 5 in CD_3COCD_3

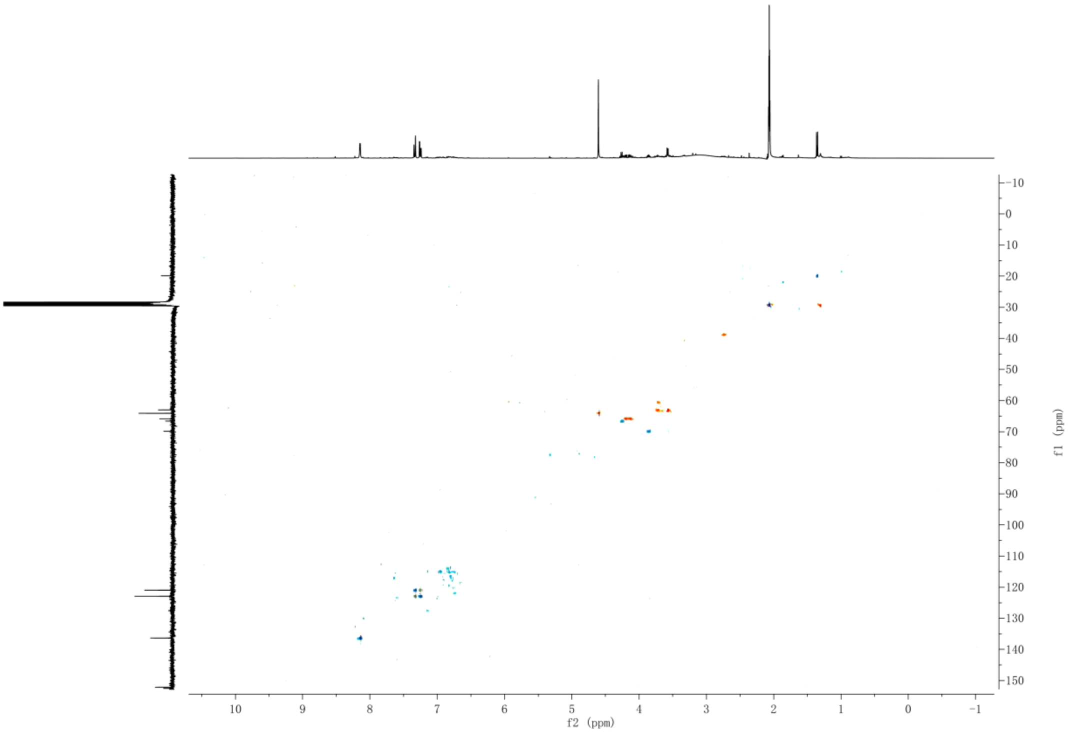

Fig. S29. HSQC spectrum of 5 in CD_3COCD_3

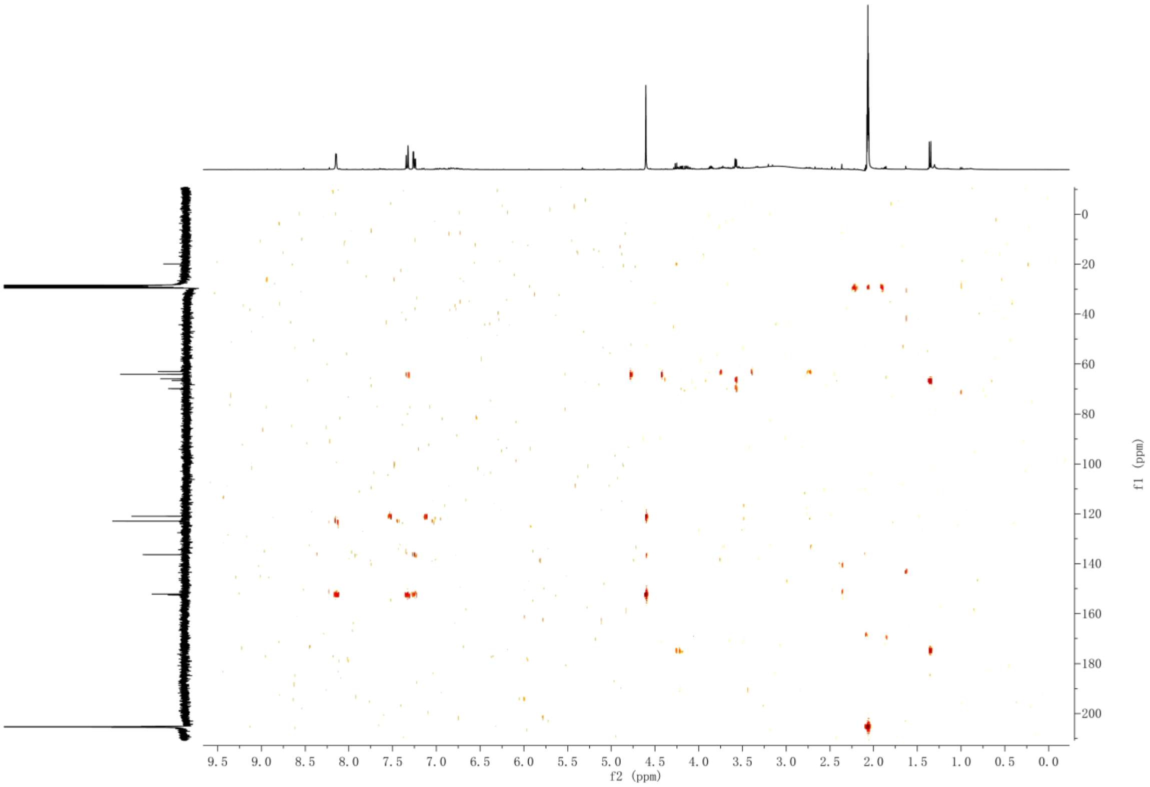

Fig. S30. HMBC spectrum of 5 in CD_3COCD_3

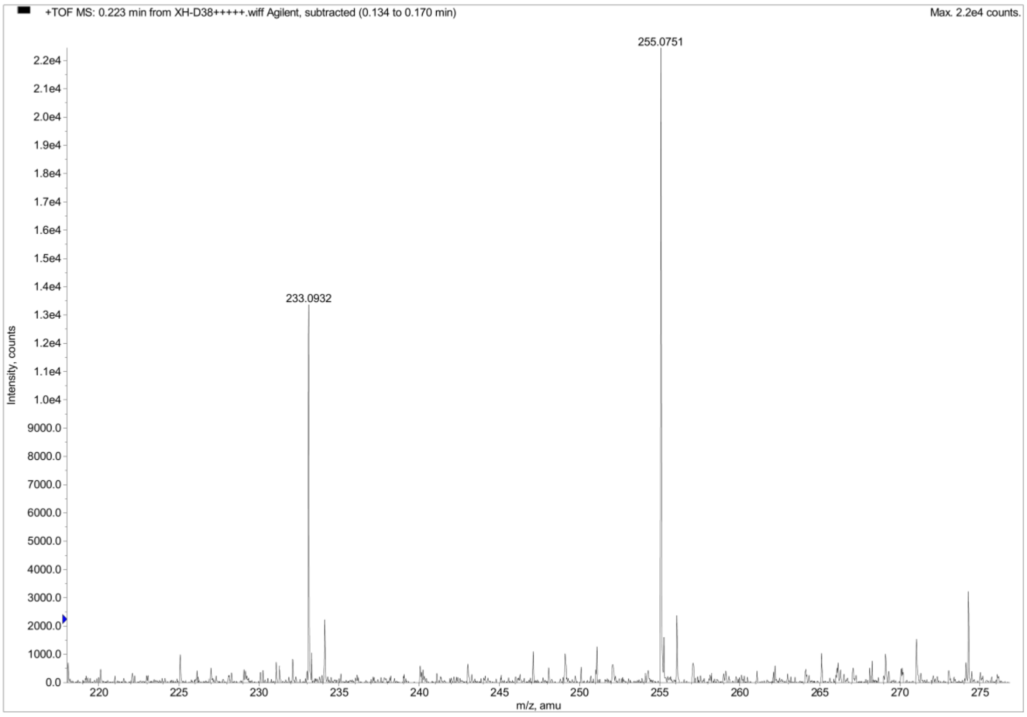

Fig. S31. HR-ESI-MS spectrum of 5
